# A Cross-Sectional Study of General Nutrition Knowledge among Nursing Students in the UAE

**DOI:** 10.1155/2024/7223610

**Published:** 2024-03-01

**Authors:** Rasha A. K. Ibrahim, Aisha N. Aldawsari

**Affiliations:** ^1^Nursing Department, Fatima College of Health Sciences, Abu Dhabi 50433, UAE; ^2^Faculty of Nursing, Damanhour University, Damanhur, Egypt; ^3^Nursing Department, Fatima College of Health Sciences, Al Ain, UAE

## Abstract

**Background:**

Adolescence is a crucial time for establishing long-lasting healthy habits, but many university students often engage in unhealthy eating behaviors. Increased independence, social influences, and mental stress all play a role in leading to poor dietary choices that can impact overall health and well-being. It is important to have a good grasp of nutrition to make informed food choices and avoid the development of chronic illnesses.

**Purpose:**

To assess the nutrition knowledge of nursing students and examine the interconnections between participants' beliefs about diet, disease, and weight management

**Methods:**

: A total of 100 nursing students participated in this descriptive cross-sectional study. Data gathering was conducted from March 2023 to May 2023 at a nursing college in the UAE. Participating students completed a self-reported validated revised general nutrition knowledge questionnaire (GNKQ-R). Descriptive statistics and Pearson's correlation were used for analysis, along with univariate and multivariate analyses as a statistical methodology to examine individual variables within the dataset independently. Cronbach's alpha coefficient of the scale in this study is 0.880. A cut-off point of 50 percent correctness was employed to signify a high level of knowledge.

**Results:**

All participating students were female, with the majority (94%) being single. Most students (68%) had a normal mass index of 23.6, whereas 32% were overweight. The analysis showed that more than half of the students (57%) rated their overall health condition as good. The analysis also found that the only variable detected to predict the good nutrition knowledge level among studied students was having children less than 18 in the same family.

**Conclusion:**

The results of the current study indicate that future wellness actions should prioritize increasing students' general nutrition knowledge while also considering individual and academic factors impacting NK in college.

## 1. Background

Lifestyle patterns tend to become entrenched in adolescence, which in turn can lead to desirable lifelong health habits [1, 2]. Adolescence is the second fastest growth period in life after infancy [3]. Proper food selection that ensures the intake of different types of nutrients, namely, macronutrients and micronutrients, is essential to support growth during this sensitive period and to lay the foundation that will last into adulthood [4, 5]. University students are most likely to be engaged in unhealthy dietary practices that consequently negatively affect their well-being [6]. Nonetheless, consuming proper nutrition is viewed as the backbone of overall health promotion, including promoting mental health as well as physical well-being and helping them keep an eye on their academic performance and increase their productivity [3, 5]. The literature has recognized the importance of the transitional periods, from late childhood to young adulthood, as it presents, indeed, a distinct critical and important period for health promotion [7, 8]. This period is critical and vulnerable to unhealthy practice adoption and body weight changes. Thus, adopting a healthy lifestyle is important [1, 9, 10].

The dramatic increase in calories needed is affected by adolescents' changing mental and social mindsets [5, 8]. Most of them become more independent and are affected by their peers, especially in their late adolescence phase when they start university time [11]. Consequently, due to those changes, all the increased nutrient needs may not be met properly [2, 12]. According to previous literature, diet rituals among university students are influenced by various personal, social, and environmental factors [13, 14]. Among the factors that are considered determinants of poor dietary behavior are knowledge deficiency in healthy food selection and fast food advertisements [15–17]. Moreover, college students' eating practices could be affected by exam stress, peer pressure, and university lifestyle [18, 19]. In previous studies, accommodation changes influenced their food choices. Another important fact is the influence of such behaviors on the occurrence of noncommunicable diseases (such as cardiovascular disease, obesity, hypertension, and cancer) [2, 5]. Dietary habits and physical inactivity are the major preventable risk factors accenting adolescence as the period for a successful prevention program [11, 20].

Adequate nutritional knowledge (NK) has been identified as gaining recognition of nutrition-related practices and concepts in terms of health disease and food intake, nutrient sources, and nutrition standards [21, 22]. Having solid nutrition knowledge is considered an inevitable factor for healthy food choices, disease prevention, and health promotion [19]. Accordingly, gaining acumen in the determinants of NK permits us to obtain a full idea of the aspects that are deficient and require improvement [7, 23]. Studying NK among female adolescents is crucial to start disease prevention earlier [24]. Additionally, possession of a good level of NK in the young adult population would facilitate the identification of those who would benefit most from nutritional education activities [25, 26].

A series of studies on university students have indicated inadequate knowledge of various nutritional topics [27–29]. In particular, questions about fruit and vegetables, milk or dairy products, food labels, and the relationship between food and chronic diseases were asked. Barzegari's research has indicated that there was a positive association between students' nutrition knowledge and dietary attitudes [11]. In another cross-sectional study at 37 universities, most of the participants mistakenly answered questions about calcium, fibre, fruits, vegetables, and dairy product intake recommendations [30].

To assess NK, the general nutrition knowledge questionnaire (GNKQ) was developed and validated by Parmenter and Wardle and is widely used in various groups and settings [31]. The GNKQ was then adopted in Australia, Turkey, China, and Japan, according to different dietary guidelines and nutritional recommendations in their countries [4, 22, 32–34]. Prior studies among college students who employed the GNKQ to assess students' knowledge have reported an average nutritional knowledge, whereas the mean score of correct answers ranged between 51% and 67% [1, 19, 22, 27, 35]. An investigation carried out in the UAE sought to authenticate the Arabic version of the GNKQ-R questionnaire among university students. The study concluded that further investigation is required to ascertain the applicability and efficacy of the Arabic GNKQ-R in older individuals and across different Middle Eastern Arab countries [36]. Another study conducted at 13 colleges within the University of Sharjah, involving teachers, staff, and students, deployed a 50-item version of the GNKQ. The study revealed that the respondents possessed insufficient information regarding making good food choices in their daily lives [23].

After thoroughly examining the available academic literature, it becomes apparent that there is a recurring pattern indicating a worrisome tendency among university students, whereby they demonstrate insufficient understanding of various aspects related to nutrition. This deficiency in nutritional knowledge carries consequences beyond the confines of academia, as it affects students' overall well-being, dietary decision-making processes, and vulnerability to diseases linked with inadequate nutrition [25, 26]. Nurses are becoming more crucial in the prevention and management of noncommunicable diseases. It is important for nurses involved in this area to have a solid understanding of nutrition and be able to apply that knowledge to their own lifestyle choices.

Internationally, extensive studies have been conducted on the nutritional knowledge of university students. However, as far as we know, there has been no research conducted on the nutrition knowledge of nursing students in the UAE. A recent review study carried out in the UAE examined published data from 2010 to 2022 to evaluate the nutrition situation [7]. The study determined that evaluating the dietary habits of the population is essential for aiding policy decision-makers in formulating and executing efficient health policies and initiatives. This study seeks to evaluate the extent of nutrition knowledge among nursing college students and identify possible characteristics that contribute to a high degree of understanding in nutrition.

## 2. Materials and Methods

### 2.1. Design and Setting

This is an institution-based cross-sectional, descriptive study that investigated nutrition knowledge (NK) among nursing college students and identified predictors of good NK. The study took place at Nursing College in the academic year 2022-2023. Nursing students at all undergraduate levels were included in the study population. The number of participating nursing students was calculated using Epi-Info program version 7 to account for a 5% margin of error, 95% confidence level, and 0.80 power at a 0.5 significance level by considering a 5% nonresponse rate. The final sample size was 100 nursing students who were available and agreed to participate at that time.

### 2.2. Study Instruments

Data were collected by using a validated revised general nutrition knowledge questionnaire (GNKQ-R). According to [22], the GNKQ-R scale consisted of 88 items under four dimensions [22]. Cronbach's alpha coefficient test was employed to assess the internal consistency of the items and evaluate the reliability of the study tool. The instrument had a reliability coefficient of 0.880, which was statistically significant at a significance level of *P* < 0.05. The four sections were depicted as follows: dietary recommendations (18 items; *α* = 0.705), nutrient sources of foods (36 items; *α* = 0.707), healthy food choices (13 items; *α* = 0.733), and diet-disease and weight management (21 items; *α* = 0.787). The subsections of the question are treated as separate items.

All questions were equally weighted (0 = incorrect or missing answer, 1 = correct answer). Demographic and academic-related questions were included at the end of the GKNQ-R survey. Among the collected demographic data were gender, marital status, perception of health, studying a nutrition course, and living with children under 18 years old. The researchers computed the average score for each section and the overall scale score which was the average of the four dimensions; higher scores indicated a good NK score ranging from 0 to 88. Cronbach's alpha coefficient test was used to measure the internal consistency of items to check the reliability of the study tool, which was found to be reliable at a statistical significance level of *P* < 0.05 (*α* = 0.880). Ten percent of the participants (*n* = 10) from the context specified above participated in the pilot study to evaluate the clarity and utility of the instruments, identify potential obstacles during data collection, and determine the length of time required to complete the tools. The pilot study participants were not included in the study sample.

### 2.3. Data Collection

An e-mail was sent to students with a link to the survey questionnaires. Attached to the e-mail was the letter of information that explained the goal of the study, the advantages to the participants, and their rights as participants. It was also emphasized in the e-mail that participation was voluntary. Each survey took approximately between 10 and 15 minutes to complete. Some students contacted the researchers for clarification, and all confusion regarding questions was resolved. The data were gathered over the course of a period of three months, beginning in March 2023 and ending in May 2023. There were no missing data because the data collection was completed after the calculated sample size (*n* = 100) was attained.

### 2.4. Ethical Considerations

The study protocol, research tool, and consent forms were presented to the Research Ethics Committee at Fatima College of Health Sciences in the UAE for evaluation. The committee granted ethical approval for this study under reference number (FECE-2-03-23-NUR-Rasha). The participants voluntarily engaged in the study after being informed about the steps taken to safeguard the confidentiality of their data. The input provided by the respondents was completely anonymous, without any recorded personal information. The goal and objectives of the research were effectively communicated to the nursing students participating in the study through the dissemination of information sheets and consent forms.

### 2.5. Statistical Analysis

The data were analyzed using SPSS 20. Descriptive statistics (frequency, means, standard deviations, minimum and maximum, and percentages) were deployed to quantify demographic variables. The normality of the data was determined using the Shapiro‒Wilk test. The sample was found to be normally distributed, and the null hypothesis was accepted for the GNKQ-R (*P* value ≥0.001). Cronbach's alpha coefficient verified that the data reliability with values above 0.7 is acceptable [37]. The study exploited Pearson's correlation coefficient to examine the connections between diet-disease and weight management, along with the factors of dietary recommendations, food group, and healthy food choices. All statistical tests were performed at a significance level of 0.05. Following this, the usage of univariate analysis served as a statistical methodology to independently investigate individual variables within the dataset. This analysis aimed to explore the distribution and characteristics of each variable in isolation, thereby facilitating the identification of patterns, trends, and significant factors that may influence the outcome of interest. For the purpose of analyzing the data, a response rate of 50% or more for each question is deemed to represent a good degree of knowledge, while a score below 50 indicates a poor level of knowledge.

## 3. Results and Discussion

The study participants' characteristics show that 100% of the participants were female, with the majority (94%) being single. Most students (68%) had a normal body mass index (BMI) of 23.6 ± 12.97, whereas 32% were overweight, with a mean that was greater than 25 kg/m^2^. Slightly more than half of the students (57%) rated their overall health condition as good, with more than one-third (37%) perceiving it as poor/fair. In the entire sample, 45% of the subjects were enrolled in nutrition courses, and 55% were not enrolled in nutrition courses. Almost four-fifths (83%) of students were part of families with children younger than eighteen Table 1.

As indicated in Table 2, nursing students' overall general nutrition knowledge was good (73%), with a mean percent score of 53.86 ± 19.44. For the individual subscales, “food groups” had the greatest mean score (57.53 ± 13.65), followed by diet-disease and weight management (53.86 ± 19.44), whereas “healthy food choices” had the lowest mean score (51.08 ± 22.61).

Table 3 shows that a significant positive correlation was found between nursing students' perceptions of diet-disease and weight management and each (dietary recommendations, food group, and healthy food choices) as *P*≤0.001^*∗*^. Additionally, the knowledge of healthy food choices of general nutrition knowledge had a higher correlational value of diet-disease and weight management (*r* = 0.681), while the dietary recommendations section had the lowest correlational value (*r* = 0.498).

The univariate analysis was deployed with all sociodemographic variables. The only variable detected to predict the good nutrition knowledge level among studied students was having children less than 18 in the same family. The remaining characteristics are not determinates; for simplicity in data presentation, they have been excluded from the table. Positive coefficient values indicate a positive influence on the NK score (Table 4).

## 4. Discussion

Students can improve the quality of their diets for every phase of their lives by acquiring a profound understanding of nutrition and embracing healthy eating practices; therefore, checking students' current levels of knowledge provides more effective plans in higher education settings. This study aimed to examine nutrition knowledge levels among nursing college students and plausible determinants of good NK. Our findings showed that the majority of participants were single, female, and undergraduate students, similar to previous literature [1, 10, 21, 38].

Our results demonstrated that most students had normal BMI (Table 1). A similar pattern of results was obtained in other literature [1, 11, 21, 25, 38, 39]. Other studies found the opposite; for example, in China, Chen et al. found a lower number of overweight female students (11.9%) [26]. On the other hand, a study conducted by Ul Haq et al. [24] reported that international students had a higher prevalence of overweight and obesity.

In the current study, it is noticeable that the majority of students had normal body weight, even though 37% perceived their health as poor/fair. In the same vein, Belogianni K et al. reported that 30.5% of students rated their health as poor [1]. We speculate that this might be because 55% of students are not enrolled in nutrition courses and they do not have sufficient knowledge about good health characteristics. Another justification is that they overestimate the health they are aiming to achieve.

The study shows that the overall NK in the sample was 53.86, which corresponds with the results of the study conducted on 378 male students at Kuwaiti University, as they revealed that the mean percentage of overall NK in the sample was 56.9% [18]. In comparison to the current study results, a study conducted with 253 participants reported a high overall level of NK among students [21]. A probable explanation for having an excellent grasp of nutrition knowledge is the fact that most of the sample came from health backgrounds that may have included a nutrition course; nevertheless, in the present research, more than half of those participating had never studied nutrition previously. However, additional research involving undergraduates in Australia's dance program, New Zealand's nursing program, and Ghana's nursing program found low overall nutritional scores [27, 28, 30].

The data revealed that food groupings received the greatest score, while healthy food choices scored the lowest grade. In a comparable manner, a research study was conducted in the United Kingdom to investigate the level of NK among university students [1]. These results are higher than those reported in a prior study [35] that used the same tool version. This could be attributable to the ease with which knowledge is available in our study context, as well as the broad nutritional awareness campaigns throughout the country. A positive correlation was detected between overall nutrition knowledge and attitudes toward weight management and decreasing diet-related health issues. This finding is consistent with a recent study that found a positive and substantial relationship between health-related performance and nutrition knowledge [40].

Surprisingly, being a member of a family with children under the age of eighteen was the only predictor for identifying characteristics predicting healthy nutrition levels among nursing students. Likewise, the preliminary evaluation of the Identification and Prevention of Dietary- and Lifestyle-induced Health Effects in Children and Infants study included 1435 families from eight European nations. The study demonstrated that the domestic food environment has a greater influence on children's consumption of nutritious foods compared to unhealthy foods, especially among younger children [41]. A study conducted in five districts in Oman revealed a strong correlation between the nutritional intake of children aged 6–10 years and the dietary and exercise habits of their families [42]. This has been ascribed to the implementation of numerous programs and initiatives in United Arab Emirates schools aimed at improving the eating habits of children, which have an indirect impact on family diet choices. Another justification is that, according to school policy, bringing nutritious food from home is utilized as a motivator for children; for example, if the child brings no junk food to school, they are awarded points. As a result, this strategy has been reflected in the general level of family knowledge.

While the current study found that 45% of individuals (Table 4) were enrolled in nutrition courses, this was not a predictor of having a high degree of nutrition knowledge (*P*=0.403), similar to other literature studies [5, 32]. This finding, on the other hand, contradicts earlier findings suggesting that students studying nutrition or from health sciences backgrounds had higher NK than those from nonmedical fields [21, 22, 28]. We are startled by this finding; however, we assume that it is because those who participated in the study were only enrolled in a nutrition course for a few weeks and did not have enough information about the entire course.

## 5. Conclusion

In the hospital system, nurses play a crucial role in influencing patients, particularly regarding their nutritional state. Thus, nurses should possess the necessary abilities and knowledge of nutrition. In addition, it is crucial that nursing students graduate with a strong understanding of nutrition. This study found that nursing students are quite knowledgeable about nutrition. More significantly, it was discovered that participating nursing students with younger family members had higher nutrition knowledge levels. Our research indicates that nursing students are well prepared to educate their patients about nutrition in their future nursing careers. According to this study, the nursing program is effectively equipping incoming nurses to deal with nutrition-related concerns in their clinical settings.

### 5.1. Implications

If nursing students have a strong understanding of nutrition, it may be that nursing courses effectively include nutrition teaching. To give their patients the best care possible, nurses must stay current on the most recent nutrition studies and recommendations. If nutrition knowledge is well developed among nursing students, this emphasizes the value of continuing education and training to ensure that nurses stay up to date with the most recent advancements in nutrition research and practice.

### 5.2. Future Research

To explore the specific areas of nutrition knowledge where nursing students excel and to determine whether this level of knowledge translates into improved patient outcomes in terms of nutrition status and overall health. It would also be important to study how nutrition courses could prepare those students to have a high level of nutrition. It could also be interesting to investigate whether there are any differences in nutrition knowledge between nursing students and other healthcare professionals or to explore the impact of nutrition education in clinical practice.

## Figures and Tables

**Table 1:** Sociodemographic facets of research participants (*n* = 100).

Sociodemographic characteristics	No	%
Marital status		
Single	94	
Married	6	
Body mass index (BMI)		
Underweight/normal BMI	68	68
Overweight	32	32
Mean ± SD	23.6 ± 12.97	
Perception of health		
Poor/Fair	37	37
Good	57	57
Very good	6	6
Are you studying a nutrition course?		
Yes	45	45
No	55	55
Do you have any children, under 18 years, living with you?		
Yes	83	83
No	17	17

**Table 2:** Mean scores percent score of general nutrition knowledge questionnaire-revised (*n* = 100).

Study variables	Mean % score	Poor (<50%)		Good (≥50%)	
	Mean% ± SD	No	%	No	%
General nutrition knowledge questionnaire-revised					
Dietary recommendations	51.89 ± 13.86	36	36	64	64
Food groups	57.53 ± 13.65	19	19	81	81
Healthy food choices	51.08 ± 22.61	40	40	60	60
Diet-disease and weight management	53.86 ± 19.44	32	32	68	68
Overall GNKQ-R	53.86 ± 19.44	27	27	73	73

**Table 3:** Relationship between perception of diet-disease and weight management and GNKQ (*n* = 100).

General nutrition knowledge questionnaire-revised (GNKQ-R)	Diet-disease and weight management	
	*r*	*P*
Dietary recommendations	0.498^*∗*^	<0.001^*∗*^
Food groups	0.617^*∗*^	<0.001^*∗*^
Healthy food choices	0.681^*∗*^	<0.001^*∗*^
Overall nutrition knowledge	0.642^*∗*^	<0.001^*∗*^

*r*: Pearson coefficient = small (*r* = 0.1); medium (*r* = 0.3); large (*r* = 0.4); and bigger (>0.4). ^*∗*^: statistically significant at *P* ≤ 0.05.

**Table 4:** Univariate and multivariate regression analysis to identify factors of GNK (*n* = 100).

Variables	Univariate analysis		^#^Multivariate analysis	
	*P* value	OR (LL–UL 95% CI)	*P* value	OR (LL–UL 95% CI)
Health perception	0.355	1.565 (0.605–4.047)	—	
Do you have any children, under 18 years	<0.001^*∗*^	44.296 (5.685–345.138)	—	—
Have nutrition-related qualifications	0.403	0 0.685 (0.283–1.663)	—	—

OR: odds ratio. ^#^: all variables with *P* < 0.05 were included in the multivariate. CI: confidence interval; LL: lower limit; UL: upper limit. ^*∗*^: statistically significant at *P* ≤ 0.05.

## Data Availability

The dataset gathered and analyzed in the current work is not accessible to the general public; however, it is obtainable from the corresponding author given adequate justification upon request.

## Additional Points

*Limitation*. This study was conducted among nursing students with a small number of students at one single nursing college. Therefore, generalization is not feasible.
